# Semi-supervised learning for somatic variant calling and peptide identification in personalized cancer immunotherapy

**DOI:** 10.1186/s12859-020-03813-x

**Published:** 2020-12-30

**Authors:** Elham Sherafat, Jordan Force, Ion I. Măndoiu

**Affiliations:** Computer Science and Engineering Department, University of Connecticut, Storrs, CT 06269 USA

**Keywords:** Machine learning, Positive-unlabeled learning, Somatic variant calling, Peptide identification, Exome sequencing, Tandem mass-spectrometry

## Abstract

**Background:**

Personalized cancer vaccines are emerging as one of the most promising approaches to immunotherapy of advanced cancers. However, only a small proportion of the neoepitopes generated by somatic DNA mutations in cancer cells lead to tumor rejection. Since it is impractical to experimentally assess all candidate neoepitopes prior to vaccination, developing accurate methods for predicting *tumor-rejection mediating neoepitopes* (TRMNs) is critical for enabling routine clinical use of cancer vaccines.

**Results:**

In this paper we introduce *Positive-unlabeled Learning using AuTOml* (PLATO), a general semi-supervised approach to improving accuracy of model-based classifiers. PLATO generates a set of high confidence positive calls by applying a stringent filter to model-based predictions, then rescores remaining candidates by using positive-unlabeled learning. To achieve robust performance on clinical samples with large patient-to-patient variation, PLATO further integrates AutoML hyper-parameter tuning, classification threshold selection based on spies, and support for bootstrapping.

**Conclusions:**

Experimental results on real datasets demonstrate that PLATO has improved performance compared to model-based approaches for two key steps in TRMN prediction, namely somatic variant calling from exome sequencing data and peptide identification from MS/MS data.

## Background

Personalized cancer vaccines are emerging as a promising alternative to nonspecific treatments such as chemotherapy in the management of advanced cancers [1, 2]. This approach harnesses the power of the patient’s own immune system to attack cells that express immunogenic peptides called *neoepitopes*. Neoepitopes are generated as a result of somatic DNA mutations that arise in cancer cells, hence making the immune response tumor-specific. However, only a small proportion of the potential neoepitopes lead to tumor rejection [3–6]. Methods for predicting *tumor-rejection mediating neoepitopes* (TRMNs) are the subject of much active research, including large consortium efforts such as the Tumor Neoantigen Selection Alliance [7].

Existing bioinformatics pipelines for neoepitope and TRMN prediction (e.g., [8–11]) include two main steps: (1) calling tumor-specific somatic variants from matched tumor-normal exome or whole-genome sequencing data, and (2) predicting which mutated peptides generated by non-synonymous somatic variants are presented to the immune system by the Major Histocompatibility Complex (MHC) alleles of the patient. Recent experimental work using a mouse tumor model [12] supports the utility of incorporating a third step, which prioritizes for vaccination the mutated peptides detected by tandem mass-spectrometry (MS/MS) in elutions of peptide-MHC complexes recovered from the surface of tumor cells.

Although many bioinformatics tools exist for each of these steps, there is still significant room for improvement. In particular, although many somatic variant callers have been developed based on diverse statistical models, agreement between them remains low [13, 14]. Key impediments to achieving consistently high accuracy with model-based methods include the large patient-to-patient variation in tumor purity and heterogeneity, sequencing library preparation artifacts, sequencing errors, and data processing errors such as incorrect read alignment. Several machine learning methods for somatic mutation calling have been recently developed to address this challenge [15–22]. However, most of these methods adopt a supervised learning paradigm and generally require large amounts of training data.

**Fig. 1:**
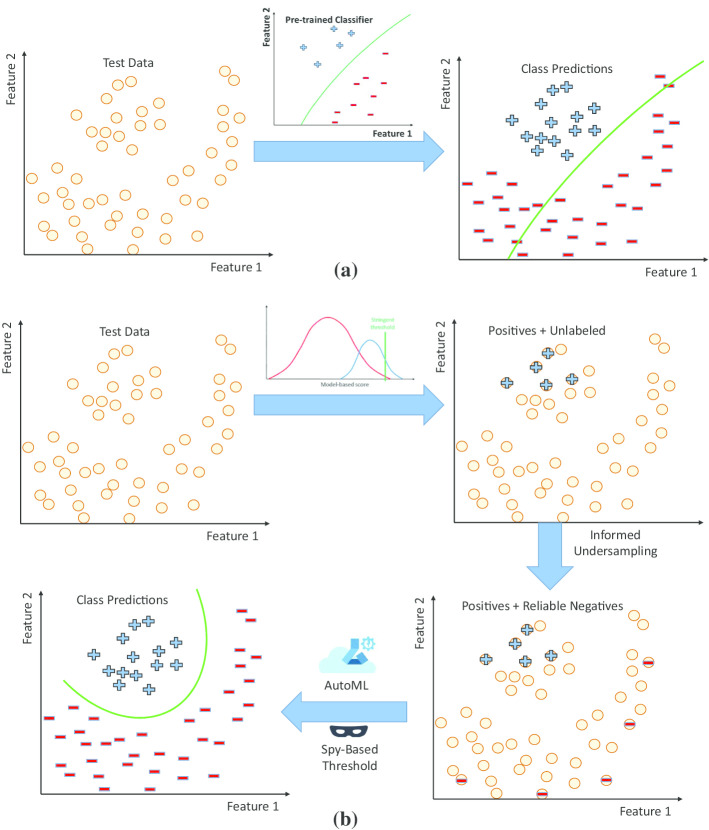
Schematic representation of supervised classification (**a**) versus PLATO’s PU learning approach (**b**). Supervised classification requires training data and can perform poorly when the distributions of training and test data do not match. PU learning uses an existing model-based classifier with stringent thresholds and informed undersampling to train a classifier from the data itself

**Fig. 2:**
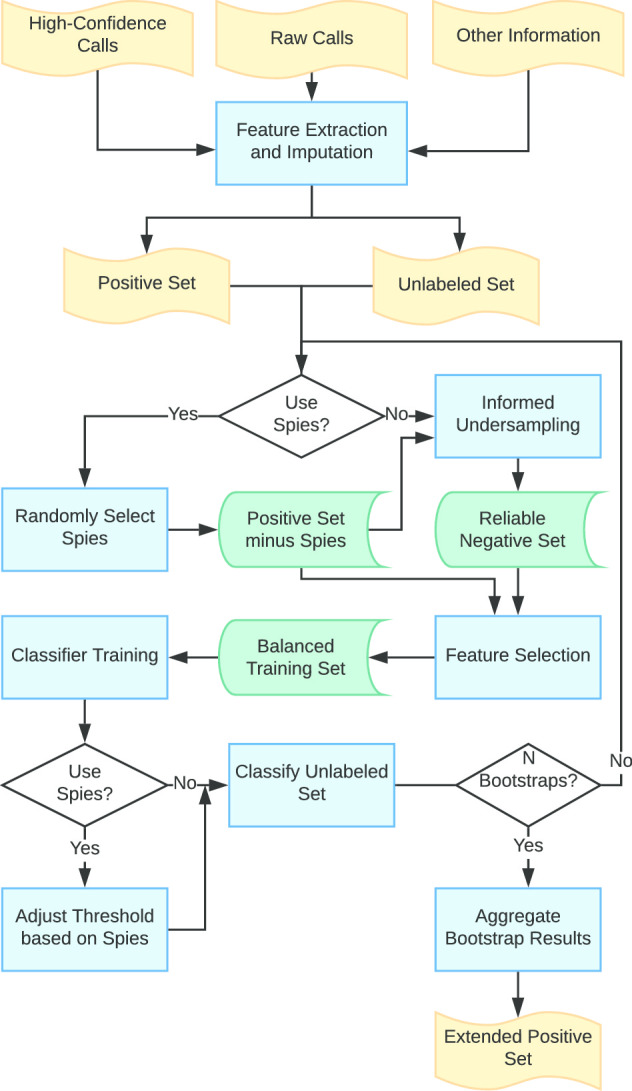
PLATO flowchart

In this paper we introduce a novel machine learning approach aimed at increasing the sensitivity of any existing model-based pipeline for somatic variant calling while maintaining a high positive predictive value. To achieve robust performance despite the significant patient-to-patient variation present in clinical samples, we adopt a semi-supervised approach that learns salient attributes from the data itself, without a need for prior training datasets. Our approach, referred to as *Positive-unlabeled Learning using AuTOml* (PLATO), is illustrated in Fig. 1 (see also the flowchart in Fig. 2). PLATO takes as input the list of unfiltered candidate somatic variant calls generated using an existing model-based pipeline along with a subset of highly confident calls obtained by applying stringent thresholds. PLATO adopts a *Positive-Unlabeled* (PU) learning approach, in which the set of highly confident calls are used as positive examples and the remaining candidate calls are used as unlabeled examples. Real cancer datasets have typical unlabeled:positive ratios of 1000:1 or higher. The vast majority of unlabeled examples are *a priori* expected to be true negatives (sequencing errors or germline variants). PLATO takes advantage of this skewed distribution to generate likely negative datasets by informed undersampling, i.e., randomly picking points that are furthest from the positive set according to the Gower distance in a space defined by categorical and numerical features such as confidence scores and allele coverage information generated by the model-based pipeline and sequence properties extracted from the genome and alignment files. PLATO then trains a classifier to discriminate between the positive and likely negative examples, and uses this classifier to label remaining data points. Hyper-parameter tuning is performed by cross-validation using the AutoML service provided by Microsoft Azure. Additionally, PLATO uses a “spy” approach for robust classification threshold selection, and performs a user specified number of bootstraps, reporting only variants with 50% or higher bootstrap support.

**Table 1:** Variant calling performance on sequencing datasets P1-P4 generated for four ovarian cancer patients

DataSet	Caller	# calls	TP	FP	TN	FN	TPR	PPV	F1
*P1*	SNVQ	515	48	114	0	0	100	29.63	45.71
P: 65	Strelka	435	48	34	80	0	100	58.54	73.85
U: 29758	2CP	65	41	8	106	7	85.42	83.67	84.54
Reseq: 162	PLATO	587	42	6	104	10	80.77	87.5	84
*P2*	SNVQ	597	147	65	3	2	98.66	69.34	81.44
P: 187	Strelka	619	149	59	9	0	100	71.63	83.47
U: 31210	2CP	187	133	39	29	16	89.26	77.33	82.87
Reseq: 217	PLATO	449	144	43	25	5	96.64	77.01	85.71
*P3*	SNVQ	629	61	13	8	1	98.39	82.43	89.71
P: 76	Strelka	306	62	11	10	0	100	84.93	91.85
U: 30289	2CP	76	57	1	20	5	91.94	98.28	95
Reseq: 83	PLATO	429	62	3	18	0	100	95.38	97.64
*P4*	SNVQ	482	48	23	87	2	96	67.61	79.34
P: 67	Strelka	380	50	94	16	0	100	34.72	51.55
U: 30176	2CP	67	45	2	108	5	90	95.74	92.78
Reseq: 160	PLATO	490	48	7	103	2	96	87.27	91.43

## Results

### Somatic variant calling from multi-technology exome sequencing data

To assess PLATO’s accuracy we used matched normal-tumor exome sequencing data generated for four ovarian cancer patients (identified in this article as P1 to P4) using two different sequencing technologies, Illumina and Ion Torrent. The unlabeled set given as input to PLATO was generated using the *Consensus Caller Cross-Platform* (CCCP) Galaxy tool available as part of the GeNeo immunogenomics toolbox [23]. CCCP incorporates two state-of-the-art somatic mutation callers, SNVQ [24] and Strelka [25], and has the ability to process multi-technology sequencing data. Positive calls were generated by applying the 2CP filter [26] on the raw output of CCCP. 2CP requires that at least one of the two callers make a high confidence call from each of the two sequencing technologies. The only exception is when one of the sequencing technologies yields no read coverage, in which case both callers must make confident calls from the reads generated by the complementary technology. The ground truth for a subset of the predicted somatic variants was established by taking the consensus of calls made from high-depth targeted re-sequencing of amplicons generated using the AccessArray system from three or more replicates per patient of both tumor and normal tissue. The first column of Table 1 gives the number of resequenced variants for each patient along with the sizes of the *P* and *U* sets. In all cases, the resequenced set included all variants that passed the 2CP filter and for which AccessArray primers could be successfully designed using the primer design tool in GeNeo. The resequenced sets also included additional SNVs called using a random forest classifier at varying levels of bootstrap support. For each compared method we computed the number of *true positives (TP)*, *false positives (FP)*, *true negatives (TN)*, and *false negatives (FN)* relative to the set of variant calls for which the ground truth was available. The reported *true positive rate*, $TPR \,{:}{=}\,TP/(TP+FN)$, *positive predictive value*, $PPV \,{:}{=}\,TP/(TP+FP)$, and *F1 score*, $F1 \,{:}{=}\,2\cdot TPR \cdot PPV/(TPR+PPV)$, were also computed relative to the ground truth available for each method.

#### Effect of classification threshold selection and classification algorithm

The users of PLATO can choose between automatic classification threshold selection based on spies or using the underlying classifier’s default threshold (typically 0.5). Also, in principle, the PLATO framework can be used in conjunction with any supervised classification algorithm. AutoML already integrates a wide range of supervised classification methods, dynamically evaluating them on each dataset using a cross-validation approach to avoid over-fitting. However, using AutoML does come with an added computational cost. To see if this added cost is warranted, we compared the AutoML-based implementation of PLATO with a baseline implementation based on random forests.

**Fig. 3:**
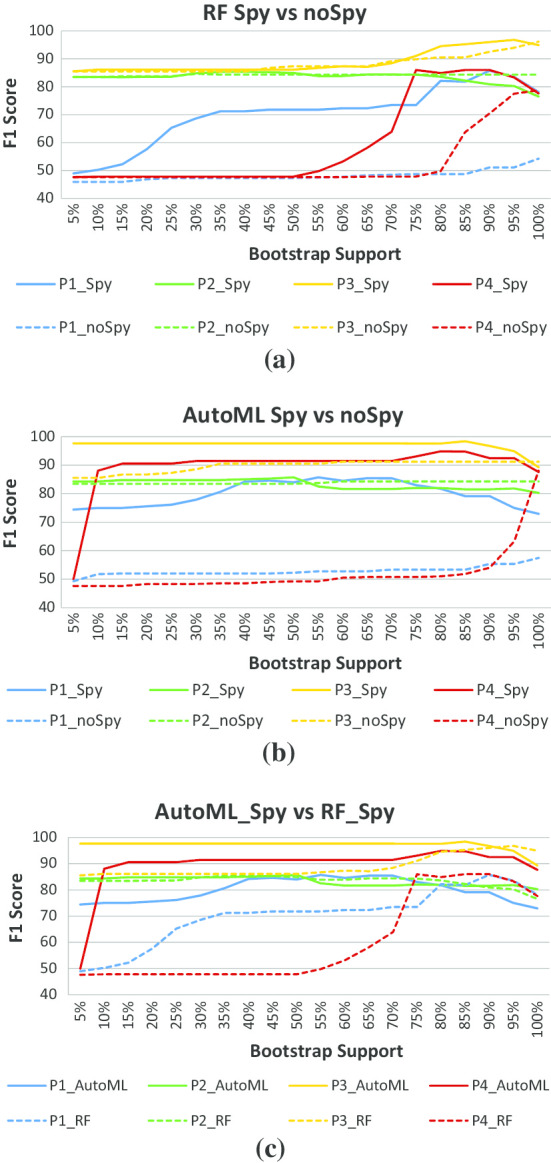
F1 scores obtained by running PLATO with $N=20$ bootstraps. **a** Random forest classification with spies-based classification threshold versus 0.5 default, **b** AutoML classification with spies versus 0.5 default, and **c** AutoML with spies versus random forest with spies. P1–P4 denote the sequencing datasets generated for four different ovarian cancer patients

Figure 3a, b show that, for both the random forest and AutoML implementations, using spies-based classification thresholds yields F1 scores close to and often better than those obtained by using the classifier’s default threshold. This holds independently of the bootstrap support required for positive classification. Furthermore, Fig. 3c shows that, when using spies-based thresholds, the AutoML-based implementation of PLATO has F1 score comparable to or better than those of the random forest implementation at virtually all bootstrap support cutoffs.

#### Comparison with model-based callers

**Fig. 4:**
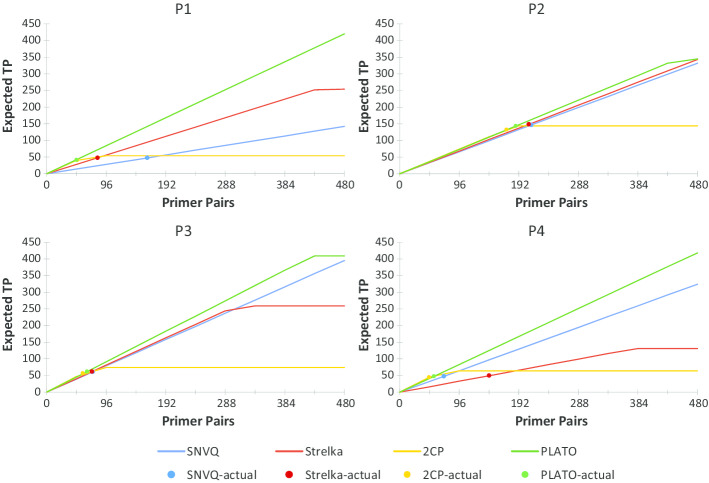
Expected TP count at different multiplexing rates for SNVQ, Strelka, 2CP, and PLATO run using AutoML, spies-based classification threshold selection, and 50% bootstrap support. The dots represent TP counts from the actual AccessArray resequencing experiment reported in Table 1. P1–P4 denote the sequencing datasets generated for four different ovarian cancer patients

Table 1 gives detailed accuracy results on the four ovarian cancer datasets, comparing PLATO with model-based callers SNVQ [24] and Strelka [25], as well as the 2CP filter of CCCP [26]. PLATO results in this table were obtained by using AutoML as classifier, spies-based classification threshold selection, $N=20$ bootstraps, and 50% bootstrap support. On all four datasets, the F1 score of PLATO is comparable to or better than that of 2CP, which in turn is comparable to or better than that of SNVQ and Strelka. Unlike SNVQ and Strelka, PLATO always retains a high PPV, comparable to or better than that of 2CP. This is important, since PLATO also makes between $2.4\times$ and $9\times$ more calls than the very stringent 2CP filter. Assuming a constant PPV this suggests that substantially more SNVs are expected to be confirmed when resequencing candidates called by PLATO on the AccessArray. Figure 4 shows for each caller the expected TP count assuming a constant PPV for up to 480 primer pairs multiplexed on a 48.48 AccessArray IFC. For reference, Fig. 4 also includes dots representing the counts from the actual AccessArray resequencing experiment reported in Table 1.

**Fig. 5:**
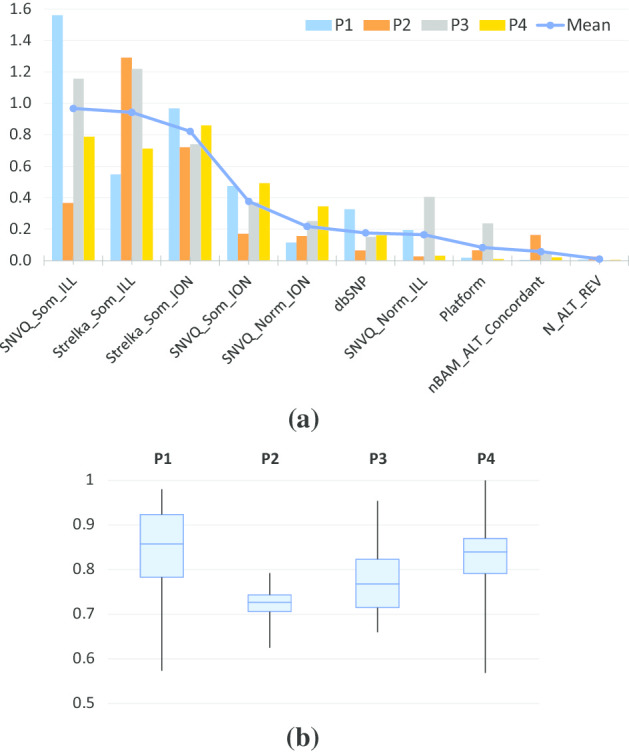
Average feature importance for SNV calling (**a**), and boxplots of the classification cutoffs selected using the spy approach (**b**) over the 20 bootstraps runs performed for the P1–P4 ovarian cancer datasets

#### Feature importance for SNV calling

Figure 5a gives the importance reported by AutoML for the top 10 features used for SNV calling, averaged for each dataset over 20 bootstrap runs (for feature descriptions see Additional file 1). Not surprisingly, the top four features are the binary somatic calls made for each sequencing technology (Illumina and Ion Torrent) by the two callers integrated in CCCP (SNVQ and Strelka). Binary calls made by SNVQ from the normal Illumina and Ion Torrent exomes and dbSNP status follow close behind in importance. The variation in feature importance from dataset to dataset is remarkably high, underscoring the need for semi-supervised methods such as PLATO that can adapt to the idiosyncrasies of each dataset. Figure 5b gives boxplots of the classification cutoffs selected using the spy approach over the 20 bootstraps runs performed for each of the four datasets. Most likely due to the over-representation of negatives in the list of CCCP candidates, the spy-based cutoffs are always higher than the 0.5 default. Furthermore, the cutoff distributions vary from patient to patient, again underscoring PLATO’s ability to adapt to each dataset.

**Table 2:** Number of peptides identified at 1% FDR from 20 MS/MS datasets generated by [27]

Sample ID	Max quant	MS-GF+	Percolator	PLATO
2014-03-04-mel3p1	2533	2967	3748	*3898*
2014-03-04-mel3p2	2770	3341	*4467*	4412
2014-03-06-mel3p1	2441	2704	3632	*3681*
2014-03-06-mel3p2	2594	3140	*4271*	4157
2014-03-04-mel4p1	2634	3108	3929	*4073*
2014-03-04-mel4p2	1765	3073	*4004*	3757
2014-03-06-mel4p1	2401	2824	3526	*3682*
2014-03-06-mel4p2	1745	2694	3856	*3954*
2014-03-05-mel5p1	3010	2517	3742	*3923*
2014-03-05-mel5p2	3342	2561	4023	*4127*
2014-03-06-mel5p1	2934	2643	3929	*4059*
2014-03-06-mel5p2	3060	2592	*4070*	4014
2014-03-05-mel8p1	3375	3057	4006	*4297*
2014-03-05-mel8p2	3764	2976	4511	*4556*
2014-03-06-mel8p1	3331	3023	4375	*4454*
2014-03-06-mel8p2	4139	3444	4801	*4839*
2014-03-04-mel12p1	1948	1601	2724	*2870*
2014-03-04-mel12p2	2013	1408	2855	*3121*
2014-03-06-mel12p1	2004	1301	*3028*	3005
2014-03-06-mel12p2	2628	1601	2942	*3356*

MHC-bound peptides were eluted from melanoma samples collected from five different patients (identified as mel3, mel4, mel5, mel8, and mel12), with two biological replicates (p1/p2) per patient, each analyzed on two independent MS/MS runs (identified by the date in the sample ID). For each MS/MS dataset, the largest number of identified peptides is typeset in italics

### Peptide identification from MS/MS data

For peptide identification, we evaluated our method on twenty datasets generated by [27] and retrieved from the ProteomeXchange repository using project identifier PXD004894. We retrieved the RAW MS/MS files for five different melanoma patients (identified as mel3, mel4, mel5, mel8, and mel12). For each patient we retrieved four MS/MS files, corresponding to two biological replicates per patient (p1/p2) and two independent MS/MS runs per replicate (identified by the date of the run, 2014-03-04/2014-03-05 or 2014-03-06). Table 2 gives the number of peptides identified at a *q*-value cutoff of 0.01 by MS-GF+, Percolator, and PLATO. Although both Percolator and MS-GF+ can compute PSM and Peptide level *q*-values, the *q*-values for all three methods were computed by our implementation of the procedure described in the Methods section to ensure that differences in peptide counts between the different methods are not due to variations in the *q*-value computation method.

**Fig. 6:**
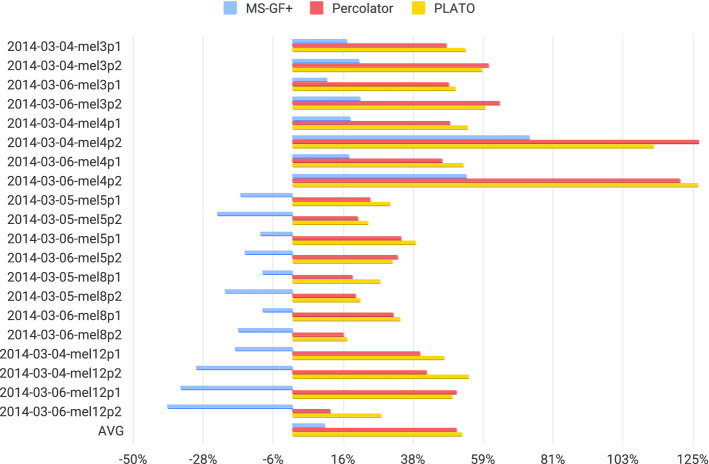
Percentage increase in the number of identified peptides over MaxQuant results reported in [27] using 1% FDR on the 20 MS/MS datasets from Table 2

For comparison, Table 2 also includes the number of peptides identified in [27] using the MaxQuant search engine with the same FDR cutoff. While we provide these numbers as a baseline, they should be considered with caution, because MaxQuant was used to search a different human proteome database. For MS-GF+ searches we used 20,585 protein sequences retrieved from Uniprot in 2019 (see Additional file 1 for details), while [27] searched a database containing 85,919 protein sequences retrieved in 2014. As shown in Table 2 and visualized as improvement over the MaxQuant baseline in Fig. 6, both PLATO and Percolator significantly outperform MaxQuant and MS-GF+ in terms of the number of peptides identified at $1\%$ peptide-level FDR. Although their perfomance is comparable, PLATO has a slight edge over Percolator, outperforming it on 15 out of the 20 datasets while being outperformed only 5 times.

**Fig. 7:**
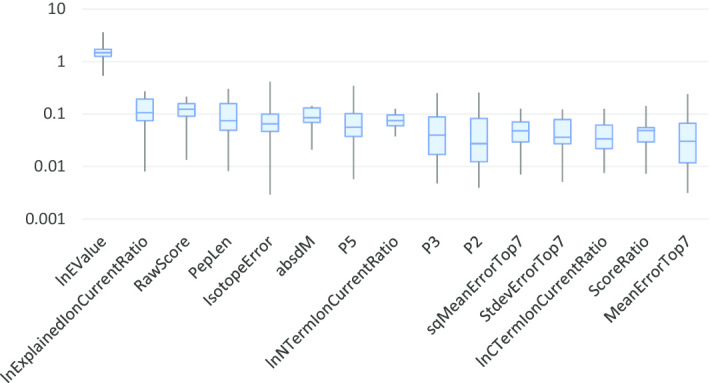
Boxplots of feature importance values (displayed on a logarithimic scale) for PLATO peptide identification experiments on the 20 MS/MS datasets from Table 2

#### Feature importance for peptide identification

The top 15 features ranked by average AutoML importance are shown in Fig. 7 (for feature descriptions see Additional file 1). The importance score of the lnEValue dominates the scores of the other features by more than one order of magnitude, and has relatively small sample-to-sample variation. Interestingly, the amino acids at known anchor positions for MHC class I binding have relatively low importance scores, most likely due to the fact that clinical MS/MS samples are comprised of peptides presented by up to six distinct MHC class I alleles, each with potentially different anchor position specificities.

## Discussion

Experimental validation results on sequencing data from four ovarian cancer patients demonstrate the effectiveness of PLATO when combined with the existing *Consensus Caller Cross-Platform* (CCCP) pipeline for somatic variant calling [23]. Since the PU learning framework is broadly applicable, we also applied PLATO to improve the rate of confident peptide identification from tandem mass-spectrometry data. Specifically, we combined PLATO with the open-source MS-GF+ database search engine [28], and used it to rescore peptide-spectrum matches (PSMs) using MS-GF+ features such as the match-score and spectrum charge, along with sequence defined features such as amino-acid composition and context. The use of PLATO increases the number of identified peptides at a fixed false discovery rate (FDR) compared to both model-based database search engines MS-GF+ and MaxQuant as well as the Percolator method, an existing rescoring approach based on support vector machines [29].

We have made available user-friendly web-based tools for peptide identification from MS/MS data by running the MS-GF+ and Percolator algorithms under the “Immunopeptidomics” section of the GeNeo Galaxy toolbox for Genomics Guided Neoepitope Prediction [8]. More information about these tools is provided in Additional file 1. A Python script that can be used to run PLATO on the output files generated by MS-GF+ is also available at github.com/esherafat/PLATO. Integration of PLATO into the GeNeo toolbox [8] is ongoing.

In future work we plan to assess PLATO’s robustness to intra-tumor heterogeneity using large-scale exome sequencing datasets such as [30] and explore further improvements in peptide identification accuracy by incorporating additional features in the PLATO search. Finally, we plan to explore supervised and semi-supervised methods for predicting TRMNs. Improving TRMN prediction accuracy is critical for enabling routine clinical use of cancer vaccines since it is impractical to experimentally assess all candidate neoepitopes prior to vaccination [31].

## Conclusion

In this paper we introduced PLATO, a novel semi-supervised approach to improving accuracy of model-based classifiers. PLATO generates a set of high confidence positive calls by applying a stringent filter to model-based predictions, then rescores remaining candidates by using positive-unlabeled learning. PLATO further integrates AutoML hyper-parameter tuning, classification threshold selection based on spies, and bootstrapping to achieve robust performance on clinical samples with large patient-to-patient variation. Although the PU-learning framework implemented by PLATO is broadly applicable, in this paper we focused on its application and evaluation in the context of two problems arising in personalized cancer immunotherapy: somatic variant calling from matched tumor-normal exome sequencing data and peptide identification from immunopeptidomic MS/MS data. This allowed us to leverage the ability to conduct experimental validation of somatic variant calls as part of an ongoing clinical trial and rely on well-established techniques for controlling false discovery rate based on template-decoy competition in the case of peptide identification from MS/MS data. Experimental results on real datasets show improved PLATO performance compared to model-based approaches for both applications.

## Methods

### Positive-unlabeled learning

Semi-supervised learning is used when available training data is a combination of labeled and unlabeled samples. The key idea of semi-supervised learning is to use the unlabeled examples to modify, refine or prioritize the hypotheses derived from the labeled data alone. Positive-unlabeled learning is an important subcategory of semi-supervised learning, where only unlabeled and positive samples are available. One popular technique for PU learning is to predict a set of likely negatives among the unlabeled samples and then apply standard supervised machine learning methods to the set of positives and likely negatives. The PU learning framework implemented in PLATO is illustrated in Fig. 2. Below we detail the key steps of this workflow.

#### Feature extraction and imputation

For variant calling, the sets *P* and *U* were generated from the output of the Consensus Caller Cross-Platform (CCCP) pipeline [23]. The set of positives was taken to be the set of variants passing the 2CP filter [26] that comes with the CCCP pipeline, and all other SNV candidates were included in *U*. Both positive and unlabeled samples were represented using a total of 110 features, 52 extracted from the output of the CCCP pipeline (see Table S3 in Additional file 1) and 58 generated using SomaticSeq [16] from the BAM files containing Illumina tumor and normal exome alignments (full list included in Additional file 1). This broad range of features included somatic variant calls made by the two somatic variant callers integrated in CCCP (SNVQ [24] and Strelka [25]), the coverage in tumor and normal samples, variant allele frequency, strand bias, membership in the list of common polymorphisms catalogued in the dbSNP database [32], average base and alignment quality, genomic region mappability, etc. The unfiltered output of CCCP includes a large percentage of missing values, typically due to low read coverage from one of the sequencing technologies. To deal with these missing values, prior to performing informed undersampling we removed the samples and features for which more than half of the corresponding entries were missing. Additionally, for the remaining samples we imputed missing features using the rfImpute function implemented by the randomForest CRAN package [33].

For peptide identification from MS/MS data, *P* and *U* were generated from the list of best *peptide-spectrum matches* (PSMs) generated using the MS-GF+ search engine for each spectrum (see Additional file 1 for details). *P* was taken to be the set of PSMs identified by MS-GF+ at a False Discover Rate (FDR) cutoff of 1% (as estimated by target-decoy competition, see below), while *U* consisted of the remaining PSMs. PLATO was run using 27 features extracted from the MS-GF+ output (see Table S4 in Additional file 1). No imputation was performed for the MS/MS data.

#### Informed undersampling and feature selection

In both of our applications (SNV calling from matched tumor-normal sequencing data and peptide identification from tandem mass-spec data) the number of unlabeled samples vastly exceeds the number of labeled positives. For example, cancer datasets have a typical unlabeled:positive ratio of 1000:1 or higher. The vast majority of unlabeled examples are *a priori* expected to be true negatives (sequencing errors or germline variants). Ideally, we would like to train a classifier that predicts with high accuracy both the minority and the majority class. However, most classifiers tend to over-predict the majority class when they are trained with imbalanced data. One solution to this issue is to generate a balanced training dataset by using undersampling.

In PLATO we use undersampling to create a balanced training dataset consisting of the positive samples and an equally-sized set of likely negatives selected from the unlabeled samples. Due to the high imbalance in the unlabeled data, randomly sampling from the unlabeled samples is likely to produce a set consisting mostly of negative samples. However, some positive samples are also likely to be picked, and the randomly selected points may not be very well-separated from positive samples in the underlying feature space. Therefore our approach is to use *informed undersampling*, where we use the positive samples to inform the selection of likely negatives from the unlabeled set. Specifically, given sets *P* and *U* of positive and unlabeled samples, we generate the set $N\subseteq U$ of likely negatives as $N=\bigcup _{i=1}^{b} E_i$, where $|E_i|=|P|/b$. Each set $E_i$ is computed by randomly selecting a batch of *m* samples from *U*, computing the average distance of each sample to the samples in *P*, and including in $E_i$ the |*P*|/*b* unlabeled samples with the greatest average distance. Since the data has both categorical and numerical features, the Gower distance is chosen as the distance measure. We chose to generate the likely negative set *N* by sampling multiple batches since the majority class might not be homogeneous (e.g., for SNV calling the negatives may represent sequencing errors or germline variants), and using multiple batches increases the chance of selecting representative samples from all regions of the majority class. For all experiments reported in this paper we used $b=10$ and $m=|P|$. We did not conduct extensive empirical evaluation of these choices, but reasoned that they provide a good tradeoff between having enough subsamples to give a good representation of the search space and keeping the computational costs low by avoiding too many pairwise distance computations.

For somatic variant calling, once a balanced training dataset is generated by informed undersampling, PLATO uses a random forest classifier to rank all extracted features and selects the features with above median rank. This feature selection approach falls under the category of embedded methods, and is often used to enhance generalization and reduce running time of subsequent model training. We chose to use random forest-based feature selection over alternatives such as unsupervised dimensionality reduction methods like Principal Component Analysis (PCA) since the method works well with both numeric and categorical features and retains interpretability. Random forest-based feature selection is also highly scalable. This is an important consideration in PLATO, which performs this step for multiple bootstrap samples to increase classification robustness, as detailed below. Performing feature selection independently for each bootstrap also reduces the risk of overfitting, as different sets of features may be selected for different bootstrap runs.

Due to the lower number of available features, no feature selection was performed for the MS/MS data.

#### Bootstrapping and spy-based cutoffs

For robustness, PLATO implements PU-learning based on informed undersampling within a bootstrapping framework and implements a scheme of automatic classification threshold selection based on spies (see the flowchart in Fig. 2). In each bootstrap iteration PLATO performs the following steps:

- Selects a set of likely negative equal to the size of 90% of positive data points using the informed undersampling method described above.
- Creates a training set by combining 90% of positive samples with the selected set of likely negatives.
- Builds a classifier using AutoML using this training set.
- Applies the classifier to the 10% of positive samples that were not included in training (“spy” samples) along with the other unlabeled samples.
- Classifies an unlabeled sample as positive, if its score is higher than the minimum score of the spy samples.

The above steps are repeated a user specified number of times (*N* bootstraps). A sample in *U* is finally classified as positive if it scores higher than the spy samples in a user-selected percentage of bootstrap runs, otherwise its final classification is negative. The idea of using “spies” was initially introduced in the text classification context [34]. As shown in the Results section, using automatically selected classification thresholds based on spies results in similar or better performance on clinical datasets than using the default classifier threshold, independent of the bootstrap support.

In general, model selection and hyperparameter tuning are complex tasks. Since exhaustively evaluating all combinations is unfeasible, we used the AutoML service integrated in Microsoft Azure to efficiently search the model space. In AutoML the user has the choice of executing an experiment on a local PC, a VM in the cloud, or a large cluster. In this work, we stored the data and executed all experiments locally. In each bootstrap of PLATO, AutoML was run on the balanced training dataset consisting of 90% of positives and an equally sized set of likely negatives generated by informed undersampling by selecting the experiment type as classification, defining the cross-validation scheme as 10-fold cross validation, and the primary metric as accuracy. For each experiment type, AutoML generates a set of initial pipeline parameters and executes a number of experiments with different parameters. In each experiment, it measures the primary metric using cross-validation and picks a new set of pipeline parameters until it reaches a threshold on execution time or the number of experiments. In the end, it builds an ensemble of different models to achieve optimal performance on the test set. Both voting and stack ensemble classifiers are currently supported. By default, they appear as the final iterations of each run. In order to have a powerful ensemble, AutoML initializes a list of up to five best scoring models (checking that their scores are within 5% of the best score) using the Caruana et al. algorithm [35]. In subsequent iterations, a new model is added to an existing ensemble only if it improves its accuracy based on the user selected metric. The voting ensemble classifier in AutoML uses soft-voting and makes predictions based on a weighted average of predicted class probabilities. The stack ensemble classifier has a two-layer implementation. It takes the same models as the voting ensemble as the first layer, and the second layer trains a meta-model to find the optimal combination of models from the first layer. The default meta-model for classification tasks in AutoML is LogisticRegression. In our experiments, the algorithms that were run through AutoML iterations included BernoulliNaiveBayes, ExtremeRandomTrees, LightGBM, RandomForest, and SGD. However, the best-fitted models selected by AutoML were always Voting and Stack ensemble classifiers. For example, in the peptide identification problem the Voting ensemble was chosen as the best model 90% of the time and the Stack ensemble was selected 10% of the time. We have chosen 10 as the number of AutoML iterations per bootstrap based on preliminary experiments showing that more than 10 iterations yield diminishing improvements in accuracy.

### Processing mass-spectrometry data and assessing the false discovery rate

RAW files were converted to MGF format using RawConverter 1.1.0.19 [36]. We used the MS-GF+ search engine [28] to search the MGF files against the human proteome, with Unspecific Cleavage, and the TDA (Target-Decoy Analysis) option turned on. The decoy database generated by MS-GF+, consisting of reversed target peptides, was concatenated with the targets for FDR estimation. The MS-GF+ MZID output files were converted to PIN (Percolator INput) format using the msgf2pin utility from Percolator. The PIN files were post-processed using Percolator as well as PLATO. Further details on MS-GF+ and Percolator settings can be found in Additional file 1.

For estimating the false discovery rate (FDR) of peptide identifications from MS/MS data we adopted the commonly used *target-decoy competition* (TDC) approach. The first decision that needs to be made when using TDC is whether or not to search the target and decoy databases separately, or to concatenate them before searching. In the latter setting, each spectrum is matched with either a target or a decoy; in this way, the targets and decoys compete to match with each spectrum. As advocated in [37], we use concatenated searches in this study. Assuming that a higher score is better, for concatenated searches the FDR at a certain score threshold *t* can be estimated as in [29, 38]:

1 $$\begin{aligned} FDR(t) = \dfrac{1 + \text {Number\,of\,decoy\,PSMs\,with\,score } \ge t}{\text {Number\,of\,target\,PSMs\,with\,score } \ge t} \end{aligned}$$

We control the FDR at level *Q* by finding the lowest score threshold *t* such that $FDR(t) < Q$, and only taking target PSMs with score greater than or equal to *t*. In this study, we controlled FDR at $1\%$.

Unfortunately, controlling the FDR at a level of $\alpha$ for PSMs does not imply control at the same level for peptides. As discussed in [39], a peptide present in the sample will, on average, be matched by a greater number of spectra than an absent peptide. To address this, if a peptide matches multiple spectra, we eliminate all but the best scoring PSM for that peptide. Once we have “uniquified” the peptides, we can apply the same *q*-value cutoff procedure as for PSMs. To ensure reproducibility and fair comparisons, we created a Galaxy tool to control FDR, publicly available at neo.engr.uconn.edu/?tool_id=FDR_custom_filter, and used it to filter the results of all compared methods (MS-GF+, Percolator, and PLATO) for which raw search results were generated as part of our empirical evaluation. More details on the precise procedure for controlling FDR at both the PSM and Peptide level as well as the Galaxy tool can be found in Additional file 1.

## Supplementary information

**Additional file 1.** Supplementary Methods, Figure, and Tables.

## Data Availability

MS/MS data analyzed in this study was downloaded from the ProteomeXchange repository using identifier PXD004894 [27]. Published Galaxy histories including runs for the 20 MS/MS melanoma datasets analyzed in this paper (grouped by patient) are available at: https://www.neo.engr.uconn.edu/u/jordan/h/bassani-mel3-public, https://www.neo.engr.uconn.edu/u/jordan/h/bassani-mel4-public, https://www.neo.engr.uconn.edu/u/jordan/h/bassani-mel5-public, https://www.neo.engr.uconn.edu/u/jordan/h/bassani-mel8-public, https://www.neo.engr.uconn.edu/u/jordan/h/bassani-mel12-public.

## Abbreviations

AutoML: Automated machine learning
CCCP: Consensus caller cross-platform
LightGBM: Light gradient boosting machine
MHC: Major histocompatibility complex
MS/MS: Tandem mass-spectrometry
PCA: Principal component analysis
PIN: Percolator input file
PLATO: Positive-unlabeled learning using AutoML
PPV: Positive predictive value
PSM: Peptide-spectrum match
PU: Positive-unlabeled
SGD: Stochastic gradient descent
TDA: Target-decoy analysis
TDC: Target-decoy competition
TPR: True positive rate
TRMN: Tumor-rejection mediating neoepitope
