# Supplementary material for: Semi-supervised learning for somatic variant calling and peptide identification in personalized cancer immunotherapy
Source: BMC Bioinformatics. 2020 Dec 30;21(Suppl 18):498. doi: 10.1186/s12859-020-03813-x (PMC7772914; doi:10.1186/s12859-020-03813-x)
Supplement: Supplementary file 1 — Additional file 1. Supplementary Methods, Figure, and Tables. [file 12859_2020_3813_MOESM1_ESM.pdf]

Semi-supervised Learning for Variant Calling and Peptide Identification in Personalized Cancer Immunotherapy
Additional File 1

MS-GF+ and percolator commands and settings

For the experiments reported in the paper we used MS-GF+ version `v2020.03.14`. Detailed command lines and parameters are provided below.

MS-GF+ indexing command

When searching against a proteome multiple times the MS-GF+ developers recommend using the BuildSA tool to index the proteome. Note that unlike the `tide-index` command in Crux, MS-GF+ indexing does not actually generate the peptide database to be searched. Rather, it builds suffix arrays for the proteins; this is why we do not specify any enzyme settings at this stage. The MS-GF+ index command we used was:

```
java -Xmx3500M -cp MSGFPlus.jar edu.ucsd.msjava.msdbsearch.BuildSA -d FASTA_INPUT -tda 2
```

MS-GF+ search settings

When running the MS-GF+ search, our pipeline used commands of the following form:

```
    java -Xmx10000M -jar MSGFPlus.jar -ignoreMetCleavage 1 -s MGF_INPUT
    -d FASTA_INPUT -tda 1 -o MZID_OUTPUT -addFeatures 1 -m FRAGMENTATION -e 0 -inst INSTRUMENT
-mod MOD_FILE
```

A summary of the MS-GF+ options is provided in Table S1.

msgf2pin settings

When running the msgf2pin utility to convert the MS-GF+ MZID output to a Percolator Input (PIN) file, our pipeline used the following command:

```
msgf2pin MZID_INPUT -o PIN_OUTPUT -F FASTA_INPUT -e no_enzyme -P XXX_ -m 1 -z
```

Table S2 gives a summary of the options used by this command. Note that we used a slightly modified version of msgf2pin, the source code of which can be found here: `https://github.com/mrForce/msgf2pin-PTM-Mass-Delta`. We added the "-z" option, so that post-translational modifications would be annotated with mass deltas, rather than UNIMOD accession codes. This is because, at one point, we were working with a version of Percolator that wasn't compatible with UNIMOD accession. Note that, although msgf2pin is part of the Percolator package, we used it as a standalone utility.

Table S1: MS-GF+ search options.

Option	Value	Description
-Xmx	10000M	Tells the JVM to set the maximum heap size to 10GB
-ignoreMetCleavage	1	A (hidden) setting to turn off methionine cleavage [1]
-s	MGF_INPUT	The MGF File to search
-d	FASTA_INPUT	The FASTA file to search
-tda	1	Create (and search) a combined target-decoy database
-o	MZID_OUTPUT	Filename of MZID output
-addFeatures	1	Include additional features that Percolator will use; see [1] for more information
-m	3	Sets the fragmentation method; in this case it's 3, since the data was generated with HCD fragmentation
-e	0	Use non-specific cleavage when creating peptides to search
-inst	3	Sets the instrument type<br>in this case, it's 3, since<br>the data was generated with a Q-Exactive instrument
-mod	MOD_FILE	A file containing the post-translational modifications to include in the search. We used Cysteine Carbamidomethylation as a fixed modification since iodoacetamide was used in the experiments of [2].

Percolator command

Percolator commands were of the form:

```
crux percolator --output-dir OUTPUT_DIR PIN_INPUT
```

We are currently using Percolator version `3.02.0` in Crux version `3.20-d57cff`.

Table S2: msgf2pin search options.

Option	Value	Description
	MZID_INPUT	Mzid file to convert to PIN
-o	PIN_OUTPUT	filename of the PIN output
-F	FASTA_INPUT	Proteome that was searched against
-e	no_enzyme	Type of enzyme used for *in silico* digestion.<br>Used no_enzyme if there non-specific digestion.
-P	XXX_	In the Mzid file, each peptide is associated with<br>at least one protein sequence. Each protein sequence has an accession,<br>and decoy proteins have an accession that starts with "XXX_"
-m	1	Maximum number of matches per spectra
-z		Displays PTM as mass delta, rather than UNIMOD Accession

[1] This setting is not mentioned in the MS-GF+ documentation. See this bug report for more information: `https://github.com/MSGFPlus/msgfplus/issues/51`

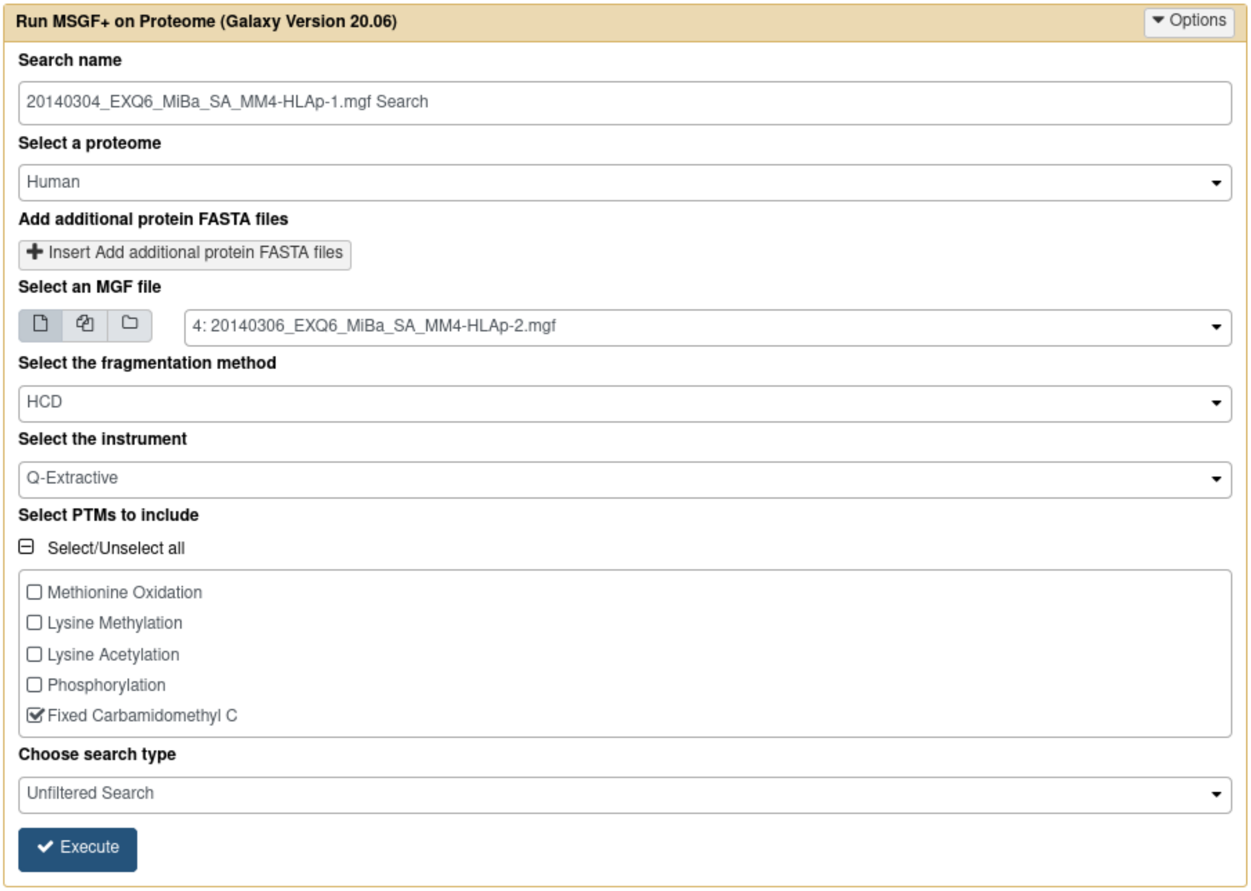

Figure S1: Interface for the Galaxy MS/MS search tool.

Galaxy Search tool

We created a publicly available Galaxy tool that allows users to run MS-GF+ and Percolator through a web-based user-friendly interface. The tool can be accessed at `https://neo.engr.uconn.edu/?tool_id=msgfplus_runner`; the tool version used in this study was 20.06. Figure S1 shows a screenshot of the interface.

The Galaxy search tool supports two search types. The first is called "Unfiltered Search", where the selected MGF file is searched against the selected proteome (concatenated with any user provided FASTA files). The second type is called "Filtered Search". Briefly the base proteome (and any uploaded FASTA files) are broken into peptides with lengths between 8 and 13 amino acids. The user specifies a set of MHC-I/HLA-I alleles, and the peptides are scored using NetMHC. For each allele-length combination, the top k percent scoring peptides are used in the search, where k is a user specified parameter. In this study, we only used the Unfiltered Search.

The user can give the search a name, and select which proteome to search. For this study, we used a Uniprot Human proteome consisting of one protein per gene, which was downloaded from `ftp://ftp.uniprot.org/pub/databases/uniprot/current_release/knowledgebase/reference_proteomes/Eukaryota/UP000005640_9606.fasta.gz` in April 2019. Currently, this is the only proteome offered for searching, though more pro-

teomes will be added in the future.

The tool has four outputs. The first is the PIN file generated by msgf2pin, except with the second row removed. This second row contains information that Percolator needs, but is otherwise not useful to us. The second output are the target and decoy PSMs scored by Percolator. The third is a log file, which is useful for debugging (and also shows exactly how MS-GF+, msgf2pin and Percolator were ran). The fourth is an archive file, which contains, among other things, the MZID output from MS-GF+, the PIN file passed to Percolator, and the output files of the Percolator run. It also contains the FASTA that MS-GF+ searched.

Published Galaxy histories including runs for the 20 MS/MS melanoma datasets analyzed in this paper (grouped by patient) are available at:

- `https://neo.engr.uconn.edu/u/jordan/h/bassani-mel3-public`
- `https://neo.engr.uconn.edu/u/jordan/h/bassani-mel4-public`
- `https://neo.engr.uconn.edu/u/jordan/h/bassani-mel5-public`
- `https://neo.engr.uconn.edu/u/jordan/h/bassani-mel8-public`
- `https://neo.engr.uconn.edu/u/jordan/h/bassani-mel12-public`

FDR Filtering

To fairly assess the number of discoveries each tool makes at a given FDR cutoff, we wrote a Galaxy tool to control FDR at both the PSM and Peptide level. The tool takes as input a tab-seperated value file, and the user specifies which columns contain the peptide, score and label (target or decoy), and the score direction (whether a bigger score is better or worse), as well as an FDR cutoff. The tool will have one output for PSM level FDR filtering, and another for Peptide level FDR filtering. For the Peptide level, it uniquifies the peptides by selecting the best scoring PSM for each peptide, and discards poorer scoring PSMs for that peptide. From then on, the procedure is the same for PSM or Peptide level FDR filtering:

```
groups ← Group PSMs by score
sortedGroups ← Sort groups by score, from best to worst
numDecoys ← 0
numTargets ← 0
endIndex ← −1
i ← 0
for score, group ← groups do
    for psm ← group do
        if psm is target then numTargets ← numTargets + 1
        else if psm is decoy then numDecoys ← numDecoys + 1
        end if
    end for
    if numTargets = 0 then
      fdr ← 1
    else
      fdr ← (numDecoys+1) / numTargets
    end if
    if fdr < α then
        endIndex ← i
    end if
    i ← i + 1
end for
```

The target PSMs in the groups up to *endIndex* are then controlled at FDR-level α. The grouping is necessary because frequently, there will be PSMs with the same score, and they must either be accepted or rejected together as a group. For Percolator, we used the "percolator score" column as the score. For MS-GF+, we used the $lnEValue$ in the msgf2pin output. This is simply the negative logarithm of a PSM's E-Value. Note that MS-GF+ provides Q-values, which can also be used for FDR control; however, their Q-values are computed based on the Spectral E-Value. The reason for this discrepancy is that we forked Percolator version 3.04 to create the custom version of msgf2pin (see the "msgf2pin settings" subsection above), and that version wasn't able to output both $lnEValue$ and $lnSpecEValue$.

As for MS/MS searches, we created a Galaxy tool that allows users to run the FDR filter through a web-based user-friendly interface. The FDR filter tool (version 20.06) can be accessed at `https://neo.engr.uconn.edu/tool_runner?tool_id=FDR_custom_filter`; Figure S2 displays a screenshot of its user interface.

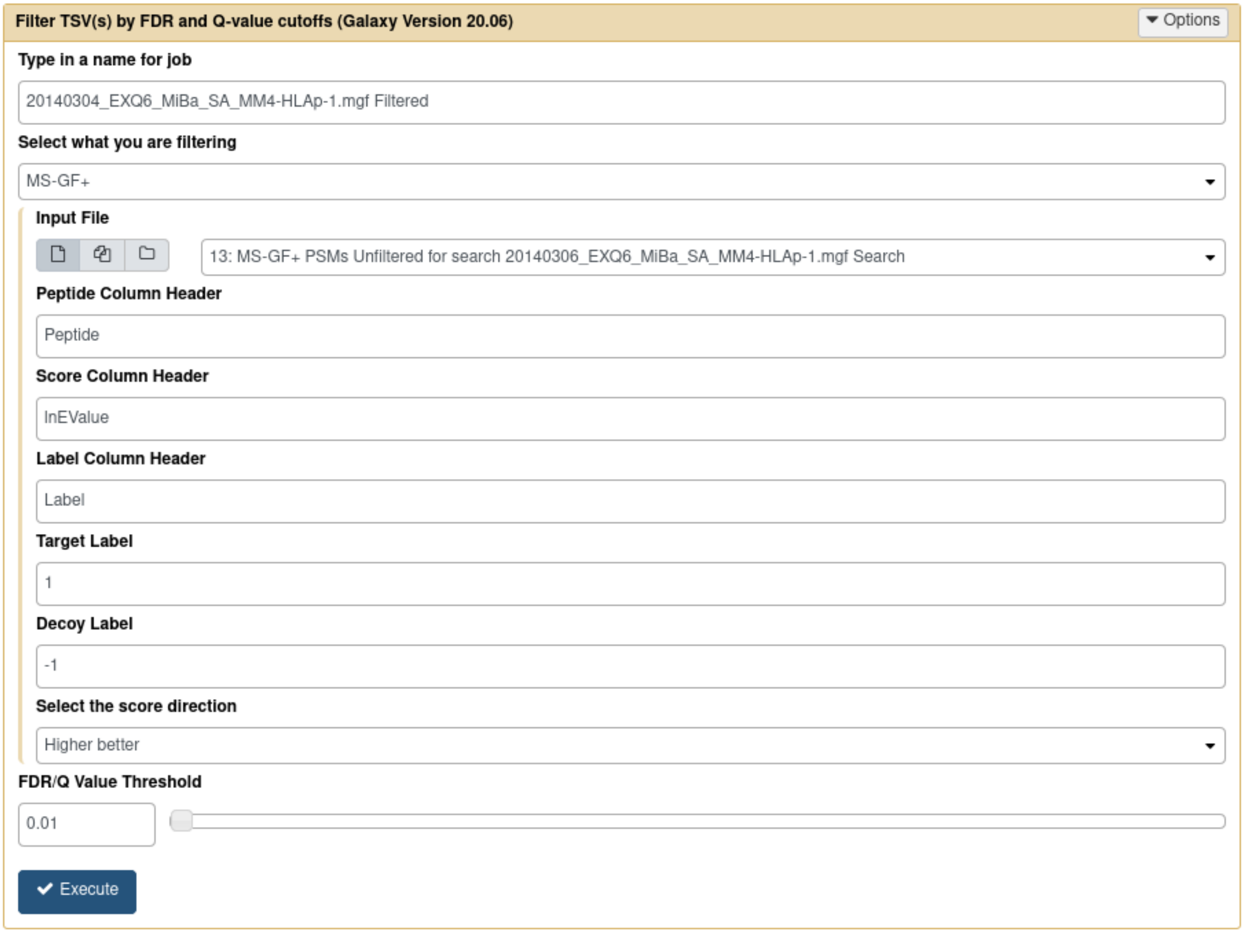

Figure S2: Interface for the Galaxy FDR filter tool.

PLATO feature descriptions

For SNV calling PLATO used 52 features generated by the CCCP pipeline (described in Table S3) along with the following 58 additional features extracted using SomaticSeq [3] from the BAM files containing Illumina tumor and normal exome alignments: Consistent_Mates, Inconsistent_Mates, MaxHomopolymer_Length, N_ALT_FOR, N_ALT_REV, N_DP, N_REF_FOR, N_REF_REV, nBAM_ALT_BQ, nBAM_ALT_Clipped_Reads, nBAM_ALT_Concordant, nBAM_ALT_Discordant, nBAM_ALT_MQ, nBAM_ALT_NM, nBAM_Clipping_FET, nBAM_Concordance_FET, nBAM_MQ0, nBAM_NM_Diff, nBAM_Other_Reads, nBAM_Poor_Reads, nBAM_REF_BQ, nBAM_REF_Clipped_Reads, nBAM_REF_Concordant, nBAM_REF_Discordant, nBAM_REF_MQ, nBAM_REF_NM, nBAM_StrandBias_FET, nBAM_Z_Ranksums_BQ, nBAM_Z_Ranksums_EndPos, nBAM_Z_Ranksums_MQ, SiteHomopolymer_Length, T_ALT_FOR, T_ALT_REV, T_DP, T_REF_FOR, T_REF_REV, tBAM_ALT_BQ, tBAM_ALT_Clipped_Reads, tBAM_ALT_Concordant, tBAM_ALT_Discordant, tBAM_ALT_MQ, tBAM_ALT_NM, tBAM_Clipping_FET, tBAM_Concordance_FET, tBAM_MQ0, tBAM_NM_Diff, tBAM_Other_Reads, tBAM_Poor_Reads, tBAM_REF_BQ, tBAM_REF_Clipped_Reads, tBAM_REF_Concordant, tBAM_REF_Discordant, tBAM_REF_MQ, tBAM_REF_NM, tBAM_StrandBias_FET, tBAM_Z_Ranksums_BQ, tBAM_Z_Ranksums_EndPos, tBAM_Z_Ranksums_MQ.

Table S3: CCCP output features used by PLATO for SNV calling.

Feature	Description
Platform	Platform supporting the call (Illumina, Proton, or Both)
Ref_allele	Reference allele
dbSNP	Yes/No common polymorphism according to dbSNP
Alt_in_dbSNP	Alternative allele in dbSNP
Alt_SNVQ_N_ILL	Alternative allele for the SNVQ call from normal exome Illumina alignments
Alt_SNVQ_T_ILL	Alternative allele for the SNVQ call from tumor exome Illumina alignments
Geno_SNVQ_T_ILL	Genotype for the SNVQ call from tumor exome Illumina alignments
Alt_Strelka_T_ILL	Alternative allele for the Strelka call based on tumor/normal exome Illumina alignments
Geno_Strelka_T_ILL	Genotype for the Strelka call based on tumor/normal exome Illumina alignments
SNV_in_SNVQ_N_ILL	Yes/No SNV called by SNVQ run on normal exome Illumina alignments
SNV_in_SNVQ_T_ILL	Yes/No SNV called by SNVQ run on tumor exome Illumina alignments
Som_SNVQ_T/N_ILL	Yes/No somatic SNV called by the SNVQ subtraction method based on tumor/normal exome Illumina alignments
Som_Strelka_T/N_ILL	Yes/No somatic SNV called by Strelka based on tumor/normal exome Illumina alignments
Total_Cov_N_ILL	Total coverage in normal exome Illumina alignments
A_Cov_N_ILL	Coverage of allele A in normal exome Illumina alignments
C_Cov_N_ILL	Coverage of allele C in normal exome Illumina alignments
G_Cov_N_ILL	Coverage of allele G in normal exome Illumina alignments
T_Cov_N_ILL	Coverage of allele T in normal exome Illumina alignments
Total_Cov_T_ILL	Total coverage in tumor exome Illumina alignments
A_Cov_T_ILL	Coverage of allele A in tumor exome Illumina alignments
C_Cov_T_ILL	Coverage of allele C in tumor exome Illumina alignments
G_Cov_T_ILL	Coverage of allele G in tumor exome Illumina alignments
T_Cov_T_ILL	Coverage of allele T in tumor exome Illumina alignments
Alt_SNVQ_N_ILL	Alternative allele for the SNVQ call from normal exome Proton alignments
Alt_SNVQ_T_ION	Alternative allele for the SNVQ call from tumor exome Proton alignments
Geno_SNVQ_T_ION	Genotype for the SNVQ call from tumor exome Proton alignments

Continued on next page

Table S3 – *Continued from previous page*

Feature	Description
Alt_Strelka_T_ION	Alternative allele for the Strelka call based on tumor/normal exome Proton alignments
Geno_Strelka_T_ION	Genotype for the Strelka call based on tumor/normal exome Proton alignments
SNV_in_SNVQ_N_ION	Yes/No SNV called by SNVQ run on normal exome Proton alignments
SNV_in_SNVQ_T_ION	Yes/No SNV called by SNVQ run on tumor exome Proton alignments
Som_SNVQ_T/N_ION	Yes/No somatic SNV called by the SNVQ subtraction method based on tumor/normal exome Proton alignments
Som_Strelka_T/N_ION	Yes/No somatic SNV called by Strelka based on tumor/normal exome Proton alignments
Total_Cov_N_ION	Total coverage in normal exome Proton alignments
A_Cov_N_ION	Coverage of allele A in normal exome Proton alignments
C_Cov_N_ION	Coverage of allele C in normal exome Proton alignments
G_Cov_N_ION	Coverage of allele G in normal exome Proton alignments
T_Cov_N_ION	Coverage of allele T in normal exome Proton alignments
Total_Cov_T_ION	Total coverage in tumor exome Proton alignments
A_Cov_T_ION	Coverage of allele A in tumor exome Proton alignments
C_Cov_T_ION	Coverage of allele C in tumor exome Proton alignments
G_Cov_T_ION	Coverage of allele G in tumor exome Proton alignments
T_Cov_T_ION	Coverage of allele T in tumor exome Proton alignments
Total_Cov_T_ILL_RNA	Total coverage in tumor RNA-Seq Illumina alignments
A_Cov_T_ILL_RNA	Coverage of allele A in tumor RNA-Seq Illumina alignments
C_Cov_T_ILL_RNA	Coverage of allele C in tumor RNA-Seq Illumina alignments
G_Cov_T_ILL_RNA	Coverage of allele G in tumor RNA-Seq Illumina alignments
T_Cov_T_ILL_RNA	Coverage of allele T in tumor RNA-Seq Illumina alignments
Total_Cov_T_ION_RNA	Total coverage in tumor RNA-Seq Proton alignments
A_Cov_T_ION_RNA	Coverage of allele A in tumor RNA-Seq Proton alignments
C_Cov_T_ION_RNA	Coverage of allele C in tumor RNA-Seq Proton alignments
G_Cov_T_ION_RNA	Coverage of allele G in tumor RNA-Seq Proton alignments
T_Cov_T_ION_RNA	Coverage of allele T in tumor RNA-Seq Proton alignments

For peptide identification PLATO used the 25 features listed in Table S4, which were extracted from the MS-GF+ output.

References

[1] Granholm, V., Kim, S., Navarro, J.C., Sjolund, E., Smith, R.D., Kall, L.: Fast and accurate database searches with MS-GF+ percolator. Journal of proteome research **13**(2), 890–897 (2013)

[2] Bassani-Sternberg, M., Bräunlein, E., Klar, R., Engleitner, T., Sinitcyn, P., Audehm, S., Straub, M., Weber, J., Slotta-Huspenina, J., Specht, K., *et al.*: Direct identification of clinically relevant neoepitopes presented on native human melanoma tissue by mass spectrometry. Nature communications **7**, 13404 (2016)

[3] Fang, L.T., Afshar, P.T., Chhibber, A., Mohiyuddin, M., Fan, Y., Mu, J.C., Gibeling, G., Barr, S., Asadi, N.B., Gerstein, M.B., *et al.*: An ensemble approach to accurately detect somatic mutations using SomaticSeq. Genome biology **16**(1), 197 (2015)

Table S4: Features used by PLATO for peptide identification from MS/MS data.

Feature	Description
CalcMass	Theoretical mass of peptide (sum of amino acid masses)
Mass	Spectrum precursor mass
dM	Theoretical mass minus observed mass
absdM	Absolute value of dM
IsotopeError	The number of additional neutrons in the peptide compared to the monoisotopic mass
MeanErrorTop7	Mean mass error of 7 most intense peaks
sqMeanErrorTop7	Square root of MeanErrorTop7
StdevErrorTop7	Standard deviation of mass errors of 7 most intense peaks
Charge1, Charge2, Charge3	Spectrum charge
DeNovoScore	Score of best scoring peptide for the spectrum. This is among all possible peptides, not just those in the database
RawScore	The PSM score assigned by MS-GF+
Energy	Difference between RawScore and DeNovoScore
ScoreRatio	Ratio of RawScore to maximum possible score (aka DeNovoScore)
lnEValue	Negative one times the natural logarithm of the database level E-value [1]. See Kim and Pevzner [4] for a detailed description of how E-value is calculated by MS-GF+
lnExplainedIonCurrentRatio	Logarithm of the total intensity of identified fragment ions divided by total intensity of all ions
lnNTermIonCurrentRatio	Logarithm of total intensity of identified N-terminal fragment ions divided by total intensity of all ions
lnCTermIonCurrentRatio	Logarithm of total intensity of identified C-terminal fragment ions divided by total intensity of all ions
lnMS2IonCurrent	Logarithm of sum of intensities of all fragment ions
PepLen	Peptide length
P1 and P6	The amino acids before and after the peptide in its protein
P2 and P3	The first two amino acids of the peptide
P4 and P5	The last two amino acids of the peptide

[4] Kim, S., Pevzner, P.: MS-GF+ makes progress towards a universal database search tool for proteomics. Nature communications **5**, 5277 (2014). doi:10.1038/ncomms6277
